# Architecture and Protocol of a Semantic System Designed for Video Tagging with Sensor Data in Mobile Devices

**DOI:** 10.3390/s120202062

**Published:** 2012-02-14

**Authors:** Elsa Macias, Jaime Lloret, Alvaro Suarez, Miguel Garcia

**Affiliations:** 1 Grupo de Arquitectura y Concurrencia (GAC), Departamento de Ingeniería Telemática, Universidad de Las Palmas de Gran Canaria, Campus Universitario de Tafira, 35017, Las Palmas de Gran Canaria (Gran Canaria), Spain; E-Mails: emacias@dit.ulpgc.es (E.M.); asuarez@dit.ulpgc.es (A.S.); 2 Instituto de Investigación Para la Gestión Integrada de Zonas Costeras, Universidad Politécnica de Valencia, Camino Vera, s/n, 46022, Valencia, Spain; E-Mail: migarpi@posgrado.upv.es

**Keywords:** video semantic sensor network, Android mobile phone, WiFi technology

## Abstract

Current mobile phones come with several sensors and powerful video cameras. These video cameras can be used to capture good quality scenes, which can be complemented with the information gathered by the sensors also embedded in the phones. For example, the surroundings of a beach recorded by the camera of the mobile phone, jointly with the temperature of the site can let users know via the Internet if the weather is nice enough to swim. In this paper, we present a system that tags the video frames of the video recorded from mobile phones with the data collected by the embedded sensors. The tagged video is uploaded to a video server, which is placed on the Internet and is accessible by any user. The proposed system uses a semantic approach with the stored information in order to make easy and efficient video searches. Our experimental results show that it is possible to tag video frames in real time and send the tagged video to the server with very low packet delay variations. As far as we know there is not any other application developed as the one presented in this paper.

## 1. Introduction

The emergence of sensors in the last 10 years has empowered the *Information and Communication Technology* (*ICT*) area [1]. Sensor networks will play a crucial role in the Internet of Things [2]. The physical values obtained by the sensors will be accessible from the Web [3,4]. Mobile and wireless communication has also grown dramatically in recent years. Nowadays there is a considerable amount of standardized wireless access options that allow the devices to access Internet resources massively [5]. Mainly because of the appearance of new admission control algorithms that guarantee QoS [6] for each user. Next generation of mobile Wireless networks 4G [7] and 5G [8] will increase the massive use of Internet resources. *Wireless Sensor Networks* (*WSN*) are always revolutionizing the way to monitor a physical system. With a WSN a lot of sensors are easily (and at low cost) deployed in a geographical area to monitor its physical properties. The spectacular growth of mobile phones sold in the last ten years has no similar precedent in the history of the business, and in some countries the number of mobile phones by person is greater than one [9]. These terminals are equipped with a powered hardware and software that allow the mobile user to practically use universal services to manage heterogeneous information and access Internet using different wireless and mobile technologies. The most prominent services are video and audio content (multimedia content). The young mobile user prefers to use *Voice over Internet Protocol* (*VoIP*) [10] or IP telephony (accessing Internet) [11] than traditional telephony services. These users usually access *podcasting, vodcasting* and *broadcatching* services in Internet.

The management of video based services is complex. A lot of information is generated and it is hard to transport this big amount of communication in the network. Recently, the hardware of video and audio is produced at low cost. This has fostered an increasing interest in multimedia WSN [12]. In [13] an excellent state of the art in Multimedia WSNs is presented. The algorithms, protocols, hardware, applications and multimedia sensors implementation are reviewed in depth. They use three different strategies: (a) Distributed video cameras connected to sensors that monitor physical properties of the monitored environment; (b) The cameras act as sensors sending directly multimedia information to a Central Server. In both cases in the Central Server there is an entity having to process the multimedia information; (c) The sensor nodes retrieve audio and video streams ubiquitously from a determined environment.

Taking into account the requirements of real time multimedia transmission and the characteristics of Multimedia WSNs, the authors in [14] and [15] propose a context-aware cross-layer based routing algorithm to maximize the multimedia content's gathering and guarantee the end-to-end video transmission delay. In [16], an energy-aware scheduling for video surveillance using Multimedia WSNs is presented. They propose an efficient scheduling of the activation and sleeping of sensor nodes in order to save energy. A particular type of Multimedia WSN, Video WSN, is presented in [17]. The authors show the problem of coverage (understanding coverage as the efficient detection of visual properties of the environment) analyzing the Field of View and Depth of View of the video sensors. They also present the need to make distributed video signal processing in the sensor nodes in order to reduce the big information volume that must be communicated in the WSN. The big amount of information produced by multimedia WSN demands the usage of semantic technologies in order to be efficiently processed. In [18] is presented a surveillance sensor application in which video sensors are connected to an Ethernet network in order to allow the efficient transport of video streams to the supervisor. The sensors use semantic information to establish direct communication among themselves.

In a semantic sensor web, as defined by Henson in [19], sensor data are annotated with semantic metadata to increase interoperability as well as to provide contextual information essential for situational knowledge. With this contextual information the raw data are enriched in order to reduce the amount of information to be stored [20]. Data from video sensors (video cameras) are complex to annotate when spatial tags are used. With these kinds of tags it is possible to position videos in geographical services like *Google Maps* or *Google Earth* or *Panoramio*. In [19] a proof of concept that consists in processing temporal semantic tags of videos stored in *YouTube* and situated the in *Google Maps* service, to seek videos using a time restriction is presented. This can be done implementing temporal tagging that allows the user to specify queries of concepts like *within*, *contains* or *overlap* [21].

In [22], Henson imagined that a mobile Android phone could capture a video and annotate it with semantic data. That mobile video could be annotated with time, place and thematic information submitted by the mobile user. Such metadata could be used to query for similar videos. From 2007 to now the evolution of mobile phones has been very important. Current smart mobile phones are provided with sensors and powerful video cameras (in some cases more than one video camera). This makes possible to realize the vision of Henson in 2007.

In this paper we present a sensor-based video tagging system that allows a mobile phone to record videos and tag them with some extra information taken from the embedded sensors. Later, the tagged video is placed on an Internet server, so it can be viewed by other users jointly with the sensed data in order to appreciate better the environment when the video was recorded. Moreover, we have added a semantic approach in the server in order to provide easy and efficient video searches. Experimental results show that videos can be tagged in real time and with a good grade of precision, and the transactions with the Web server can be done with low delays. This facilitates to implement a real time query system directly on the mobile phones in real time.

The remainder of the paper is as follows: in Section 2 we present the main concepts germane to mobile video tagging using the sensors, that are included in the current smart mobile phones, and we describe some related work found in the literature. The architecture of the semantic system, the ontologies and the semantic system operation are presented in Section 3. Section 4 is devoted to the analytical model presentation. Section 5 shows the experimental evaluation of our semantic system. Finally, in Section 6, we draw our main conclusions and future work.

## 2. Related Work

Traditionally, several formats for video tagging have been used related to different specific purposes. Moreover, the video content can be analyzed using ontology. In [23] soccer domain ontology is designed to analyze soccer videos. They use *Web Ontology Language* to define the ontology and temporal logic to describe the semantic events and reasoning. In [24], an original work on semantic video annotation is presented. In this work some interesting thoughts about this topic are discussed.

New multimedia standards, such as MPEG4 and MPEG7, provide the basic functionalities in order to manipulate and transmit objects and metadata. Concretely, MPEG7 (the formally called *Multimedia Contend Description Interface*) helps describe video content and thus enables the user to easily perform many tasks such as find, store and locate a video [25]. It has the ability to describe the low level, semantic and structural aspects of the video. Let us note that by using an automatic tool, or manually, the video’s content can be described in MPEG7, but we are not interested in describing the video’s content. We are interested in augmenting the expressivity of a video with other information sensed by other sensors. In this way, the video information can be interpreted taking into account this information. For example, different parts of a video of a beach, tagged with temperature values provided by a sensor, could enrich the video allowing the user to interpret the scene.

In [26], a very interesting survey about the state of the art in MPEG7 based ontologies is presented. In this work, the two main annotation dimensions prevailing in the literature (content structure descriptions and linking with domain ontologies) are compared.

In addition to MPEG7 a framework must be used in order to fill the gap between semantic and reasoning. In [27], the application of semantic Web technologies to semantic video retrieval is presented. The outputs are represented in MPEG7 and Web Ontology Language.

Semantic video indexing is the first step towards automatic video browsing, retrieval and personalization. It consists of two sub-processes: temporal segmentation of the video stream and semantic labeling of the resulting video segments. The objective of this segmentation is to extract video semantic units that can be associated with clear semantic meanings. In well structured videos (like TV Programs) it is possible automatically generate this semantic video indexing [28]. These semantic meanings can be structured with MPEG7 or MPEG21.

A not considered challenge is to take into account the MPEG7 annotation of videos in order to allow Peer-to-Peer communication among them as explained in [29]. Similar comments could be done for the MPEG21 standard. It is very appropriate to specify the interchange of information among users and the entire multimedia distribution chain [30]; but it is not well appropriated for our purposes.

A semantic modeling of video contents retrieval is presented in [31]. In this paper some basic ideas and concepts of semantic modeling of video (formal definition of semantic unit and association in videos) are explained. It also discusses about Query language and graphical conceptual model for video contents. This work is previous to MPEG7 standard and presents interesting basic concepts and ideas of video semantics.

In [32] a very interesting survey about automatic video annotation and ontologies for retrieving video from large data bases is presented. The authors also present challenges and applications.

In [33] a dense and good survey of video semantics is presented. This work presents models that are classified into annotation-based models and rich semantic models. The authors evaluate these two models and present twenty one rules which cover the whole process of model based video application development.

There are several ways to include semantic information from sensors. The first step is to consider a set of tags that can describe the values that the sensors provide, and the second step is to implement an ontology that can allow the extraction of knowledge.

A good introduction of the reasons for and benefits of annotating the information retrieved from sensors is done in [34]. The authors of this paper explored the benefits of augmenting sensor data with semantics; they describe the domain specific and spatio-temporal problems to be addressed. The role of knowledge representation and reasoning (Semantic Web technology), and standardization efforts underway to make sensor-related data and sensor observations widely available are shown.

In [35] the authors presented a good survey of 14 ontologies. They concluded their work saying that a combination of *OntoSensor* and the *CSIRO* ontologies represents the current limit of expressive capability for semantic sensors. But, neither current ontology nor a combination of the available ontologies is able to express all the properties required for the capabilities. So, it is needed more work in order to improve this expressivity. Moreover, the early work presented in [36] shows that it is possible the support for browsing service ontologies and the cooperative discovery of services based on an intuitive preference model, on mobile devices.

In [37] the Jena API is used for querying and inferencing over semantic representation of the data of the sensors. In this work, the authors explain the information service enabler in their *CommonSense* system and they propose an architecture for WSNs with semantic web technologies as enablers. Finally, they have tested their system on a fire accident scenario in a building.

The *Semantic Streams* framework [38] allows users to pose declarative queries over semantic interpretations of sensor data. This system takes data of several sensors placed on different locations and it allows nontechnical users to pose queries over semantic interpretations of sensor data. This framework was tested in a parking garage with three simultaneous and independent users. These tests showed that this framework allows multiple users to task and re-task the network concurrently.

In [39] we presented a development of a geo-localized mobile video application for Android mobile phones. In this paper we presented a mobile application that allows the mobile user to tag the videos recorded by the mobile and share them with other users. Because it is not easy to tag independent video frames due to the delay associated to the reading of the GPS values in the mobile phone, in the present paper we have improved the real time response of the software in order to record and store the tagged video frames in Internet servers. This process will produce a better video geo-tagging and produce new kind of queries to semantically discover properties of the full video automatically. Moreover, in the present paper we have added a semantic system in order to perform accurate searchers inside the videos.

Finally, in [40] the authors show an interesting summary about the systems that are able to tag video with GPS sensors. The systems store the video recorded with tags in order to allow semantic queries such as *where*, *when*, *who* or *hybrids*. The prototype uses a camera connected to different sensors to tag the video. Moreover, they show the difficulty of tagging video frames with sensor data. In this paper we explore the applicability of mobile phones for tagging video frames. We take maximum advantage of the mobile phone sensors in order to implement a low cost system. We will show that our proof of concept application is efficient and our experimental results show that real time video tagging (for further semantic search) could be easily used without decreasing the performance of the mobile phone.

## 3. Semantic System for Data Searching in Video Sensor Networks

In this Section we first describe the architecture of our semantic system, including a review of the tradeoffs for the design of video tagging in mobile phones. Secondly, we show the knowledge representation and finally the semantic operation of our system.

### 3.1. Architecture of our Semantic System

The implemented mobile application allows users to record videos and tagging them with semantic sensor data. This extra information is associated with each of the images composing a video recorded from a mobile phone, allowing users to exploit this information when sharing tagged videos. Specifically, the process consists in *geotaging* each physical object represented in the images that form a video and also obtain useful measurements of the mobile phone’s sensors for each image. All these data are stored in an *eXtensible Markup Language* (*XML*) file. Therefore, each recorded video file is associated with tags. The tagged video is stored in a single multimedia container that can be stored locally on the mobile phone or streamed to an Internet video Server. Then, the files can be shared with other users by accessing to the server. The users can also search for videos on that platform (Internet video Server), according to a certain semantic criteria, based on data that are in the tagged files of the shared videos.

**Figure 1.**
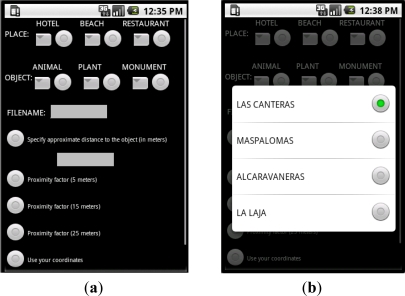
User interface of the application. (**a**) This window can be used to specify where the user is (hotel, beach or restaurant) and if he/she is focusing an animal, a plant or a monument (an object in general); (**b**) Supposing the user chose a place he/she can now specify the name of the place (in this case a beach of Gran Canaria, Canary Islands, Spain).

The *User Interface* used to add the needed data in order to begin the recording process and tagging is shown in Figure 1(a,b). As shown in this prototype application, the user must enter on *where* he/she is and *what* kind of object he/she thinks of recording. There are different options for each object type and location. The user must also specify the name of the file to be generated after the recording and tagging. And he/she must indicate the distance between the object and the mobile phone’s camera using a specific value or by choosing a particular approach factor (5, 15 or 25 m). These data will be used in the tagging process later.

Once the user has entered the above information using the user interface, then four key processes will run concurrently in order to generate tagged videos: (1) the video recording process, (2) the process of capturing the remaining data necessary for tagging from the sensors of the mobile phone, (3) the process of capturing necessary data from the *Global Positioning System* (*GPS*) and (4) the tagging process. We describe these four processes next.

The video recording process is the responsible for accessing the hardware of the phone’s camera and to capture the images that compose a video. This video is stored on the *Secure Digital* (*SD*) card in an MPEG4 file.

The process of capturing the remaining data needed for tagging accesses the sensors available on the mobile phone in order to read the sensed data which are later used to tag the video. An Android mobile phone may have a maximum of 10 sensors, which are listed next [41]:

- *Accelerometer*: It calculates the acceleration applied to the device. Specifically, it provides three values: the acceleration in the depth axis (Z axis) of the mobile phone, on the horizontal axis (X axis) and on the vertical axis (Y axis). These values are obtained by measuring the forces applied to the mobile phone and taking into account the gravity of the Earth. The three acceleration components are in *m/s^2^*.
- *Gyroscope*: It measures the angular velocity in each of the axes of the phone, in *rad*/*sec*. It is especially useful in gaming applications.
- *Light*: It detects the brightness of the screen, providing a value in SI units: *lux*.
- *Magnetic*: It measures the value of the magnetic field in the X, Y, Z axes of the mobile phone in *μTesla* unit.
- *Orientation*: It calculates three values (pitch, roll and azimuth) to detect the mobile phone inclination and the cardinal point that the mobile phone’s camera is pointing.
- *Proximity*: It calculates the proximity through the distance in *cm* between the mobile phone and the detected object. It is very useful in applications that detect human or animal presence in a given area.
- *Gravity*: It calculates the components in the Z, X and Y axes of gravity in m/s^2^.
- *Temperature*: It calculates the temperature at which the device is.
- *Rotation vector*: It represents the rotation of the device as a combination of an angle and an axis (X, Y, Z).
- *Linear accelerometer*: It indicates the acceleration along each device axis, not including gravity. All values have units of m/s^2^.
- *Pressure*: It calculates atmospheric pressure in *mbars* unit.

The GPS of the mobile phone provides two kinds of values: the geographical coordinates and the altitude of the mobile phone. Finally, the tagging process is responsible for generating a XML file that contains the information of the tagging of each of the images captured during video recording.

#### Tradeoffs of Design Related to Real Time Sensing in Mobile Phones

In the process defined above there are several factors to consider as they define the operation of the application and more specifically the video tagging. All these factors arise because the tagging should be done for each image of the video. Assuming that the mobile phone’s camera captures 25 frames per second (fps), the tagging process should be done every 40 ms. But this process is subject to the time taken by other processes (the reading of data from sensors and GPS). The time spent in reading the values of the GPS and the sensors may be higher or lower than 40 ms. Then, from now on we will assume that the average time it takes for the mobile phone’s GPS to provide a value is *T_GPS_* and the average time it takes for the sensors to provide a value is *T_Sen_* (this time is also defined for each sensor presented above). In addition, we must take into account the GPS’ reading time and capturing its values (*T_RGPS_*) and the reading time of the sensors and the time to capture their values (*T_Rsen_*). Moreover, it is also important to consider the time taken to generate the tagged file, which corresponds to the time of writing all the necessary data for each image (*T_Tag_*). We must also take into account the total processing time, which corresponds to the reading of data, its capturing, and the subsequent tagging (*T_Tot_*).

As mentioned above, we have assumed that 25 images must be generated every second (in order the user can appreciate movement). And therefore, tagged every 40 ms. But this is not entirely true and taking into account that the current video cameras use compression methods based on MPEG4, they do not generate 25 fps. Instead, they use other techniques that exploit the temporal information of a frame to generate the next (in the video sequence). Then, another factor that affects the performance is the need to synchronize the video recording and tagging effectively.

Finally, it is also important to consider the storage time of the recorded video frames. Although it is not possible to access to the code used for storage in the mobile phone we use an approximation to measure this time.

### 3.2. Ontology Approach

An ontology is a formal explicit representation of knowledge as a set of concepts within a domain, that is used to describe the domain, its properties, and the relationships between those concepts [42,43]. It provides a shared vocabulary, which can be used to model a domain. An ontology consists of classes, formed by the concepts found in the domain, and each class may have one or more parent classes. Classes may have instances, which correspond to individual objects in the domain of discourse. An ontology together with a set of individual instances of classes constitutes a knowledge base.

**Figure 2.**
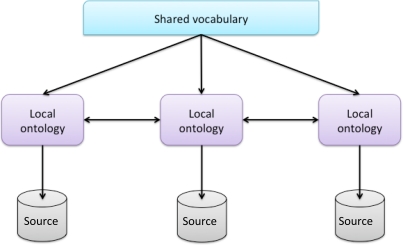
Hybrid ontology approach.

According to [44], there are different ways to employ the ontology. In general, three different directions can be identified: *single*, *multiple* and *hybrid* ontology approaches. Our system is based on a hybrid approach (see Figure 2). Each source is described by its own ontology. But, in order to make the source ontology comparable to each other they are built upon one global shared vocabulary. The shared vocabulary has primitives of the same domain.

For example, each device (mobile phone) labels the video with a GPS position, and the type of object that is being recorded, for example a flower and the user specifies if it is a rose, a poppy or a margarita. Ontology has its primitive for each flower, but we have a shared vocabulary. When we ask for a red flower, red and flower are shared vocabulary. This vocabulary must be understood by all ontologies in order to let our system be able to answer the question: Is there a video with red flower? Our system should answer with the videos of roses and poppies (red flowers), but not with the videos of margaritas (white flower). This is an easy example to explain which ontology approach is more adequate for our proposal. Now, if we introduce some more labels from the sensors of the mobile phone adequately, the system should be able to answer questions such as: in which coast is a red flower in a sunny day?

The main advantage of this approach is that new sources can easily be added without the need of modification in the mappings or in the shared vocabulary. It supports the acquisition and evolution of the ontology. Our system can be used for several applications. This scalability feature lets the system be extended without much effort. The use of a shared vocabulary makes the source ontologies comparable and avoids the disadvantages of multiple ontology approaches. The drawback of hybrid ontology approach is that it cannot be reused easily. It has to be re-developed from scratch, because all source ontologies have to refer to the shared vocabulary.

**Figure 3.**
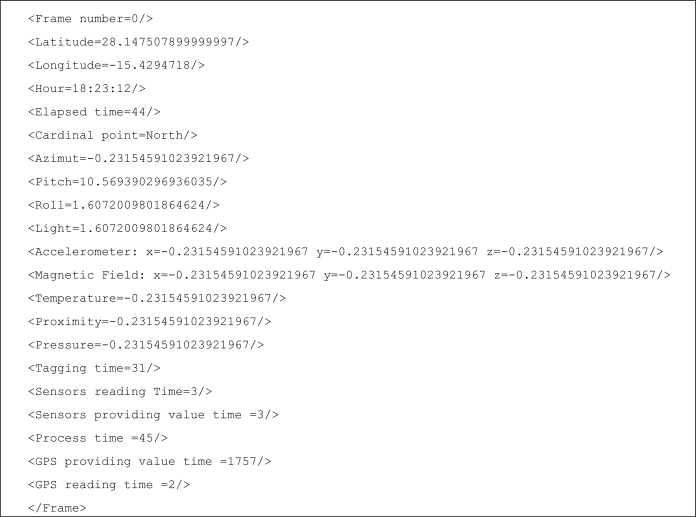
Example of video frame tagged.

An example of a part of a local ontology of our system is detailed next:

Taking into account the sensors of an Android mobile phone, we are able to generate the tagging of the video in two different levels:

1. Tagging of whole video. The idea is to provide a global description of the scene in a particular scenario. We considered the following tags:
  - <*VIDEO name*>. The video will have a name and this tag is the global one.
  - <*Place*>. Initially we have programmed for this tag only three attributes: <*Hotel*>, <*Beach*>, and <*Restaurant*> which are qualified with a name. The idea is the mobile user can specify the name of a hotel, a beach or a restaurant. This means the mobile user can be in any of these places or in a beach in which there is a hotel in which there is a restaurant. This tag can be easily extended with more attributes such as <*Mountain*>, <*Supermarket*>, <*Home*>, and many more.
  - <*Object*>. Initially we have programmed for this tag only three attributes. So, the user can specify the name of: an <*Animal*>, a <*Plant*> and a <*Monument*>. We can add many more such as <*Furniture*>, <*Food*>, <*Vehicle*>, *etc*.
2. Each frame of video (or a set of frames depending on the processing power of the mobile phone) is tagged with the values of the sensors the user wants to include. Each sensor has a tag named with the name of the sensor and an attribute that contains its value. Moreover we include the time in which the frame was captured by the video camera and the GPS value. In Figure 3 an example of tagged video frame is shown.

**Figure 4.**
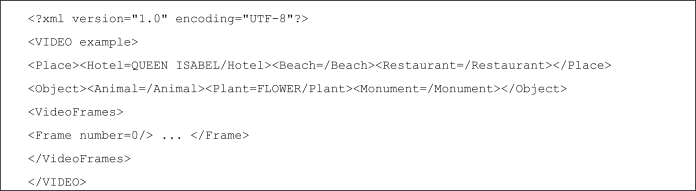
Video tagging format.

The structure of the tagging file is shown in Figure 4.

Up to now we have not added image processing for forms recognition. But our purpose is to extract concepts directly from the images. We have seen two approaches in the related literature that are being studied for our purposes: the first one uses a frame-based image classification [45], and the second one uses a Multiple Correspondence Analysis of low level features of the images [46].

### 3.3. A Semantic Search and Indexing: System Operation

**Figure 5.**
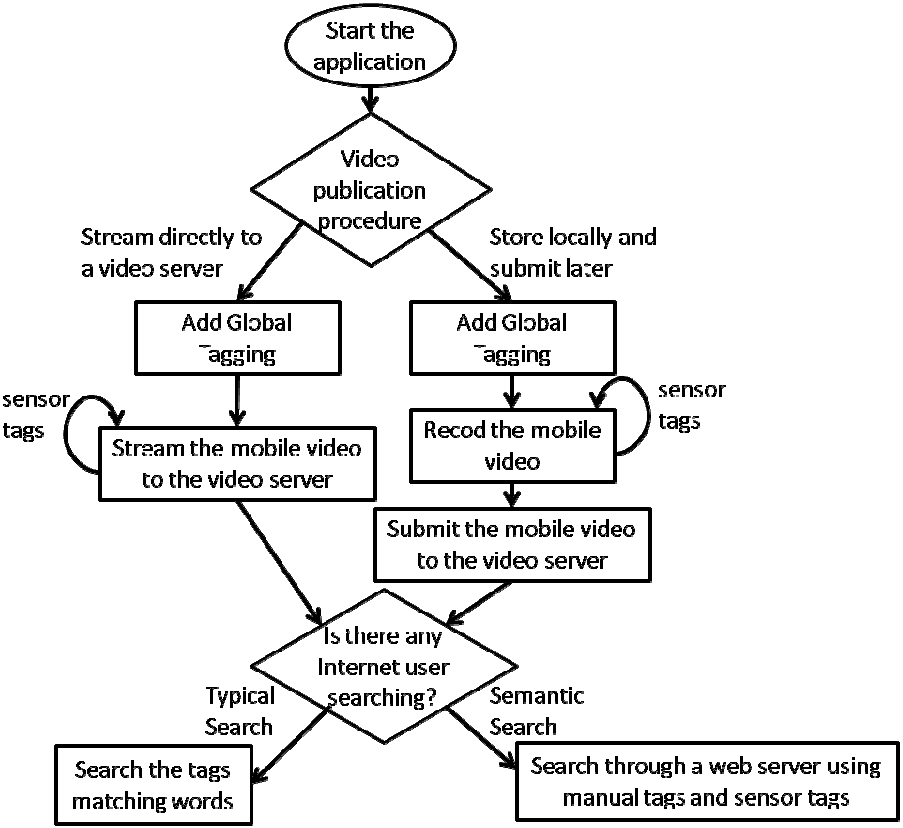
System algorithm operation.

The system operation is as follows (Figure 5): the mobile user opens the mobile application to record a video. Then, he/she introduces manually the global tags of the video and then the automatic tagging system will generate the tagging file synchronized with the video frames. There are two options to treat the tagging registering: the first one consists on saving the tagged file on the mobile phone and the second one consists on streaming it to an Internet Video Server (using an online registering). The principal advantage of the first option is that the tagging file can be done afterwards to make distributed searches over the mobile phone directly. Later, the user sends the mobile video to the Internet video Server if desires to publish it. Another advantage is that a distributed copy of the tagged mobile video could be saved in the mobile phone and in the Internet Video Server. The principal advantage of the second option is that it saves memory in the mobile phone’s memory. Because of the trend in mobile phones is to integrate enough memory, we chose the first option, but when this application is used frequently, the second option is recommended. In both cases, since the video has been annotated with several kinds of tags, the video could be saved and indexed by different Web services.

Once the mobile video has been stored in an Internet Video Server, any user (mobile or not) can search the video using a typical Web searcher or using a semantic search. Contrary to a typical Web search, a semantic searcher will admit queries like *where* (using *location* tags), *what kind of object* (using the *object* tag), *what interval of time* (using *time* tag)… For doing that, the semantic searcher uses the tag file generated when the mobile user recorded the mobile video. This semantic software can be implemented as a *proxy* or as a *plugin* that must be connected to the Web services aforementioned. Our proposal is to adapt a typical semantic searcher. When semantic searches are used, the semantic searcher must access to a service in which the mobile phones be registered. This registering software will be in charge to control the mobile phone video availability.

**Figure 6.**
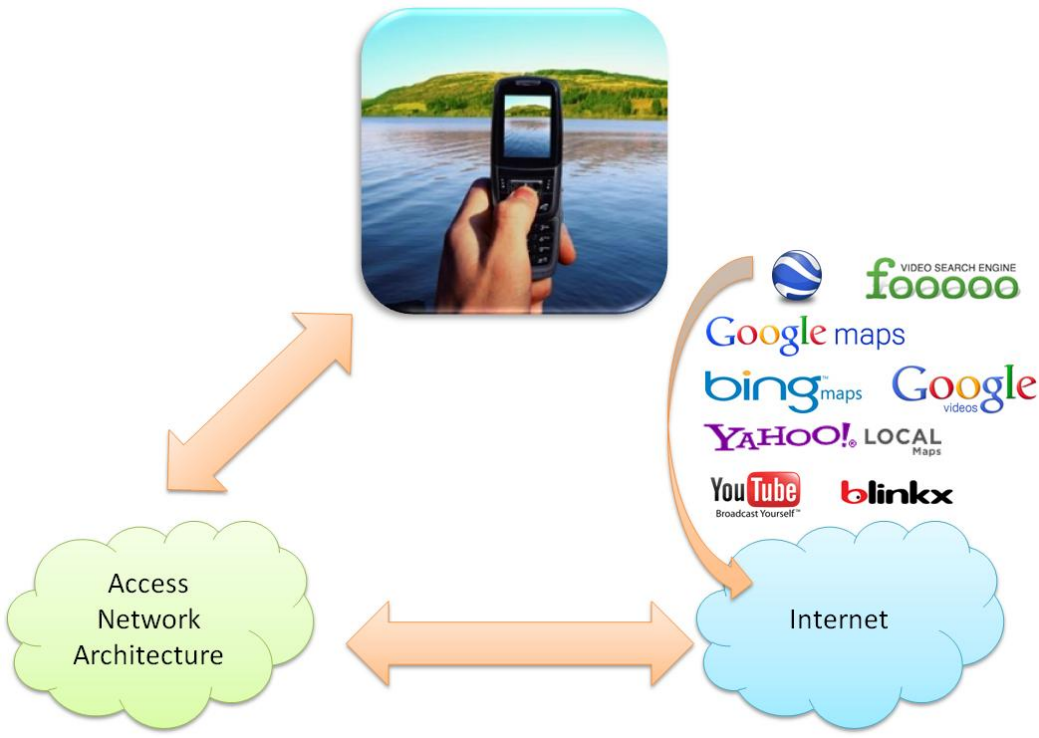
Graphic schema with an example of the operation of our system.

Figure 6 shows a schema illustrating the process. The mobile phone is connected to a wireless access network. The performance of the wireless network must support the tagged video transactions in real time. Moreover, it must be able to access the Web services specified in the figure in order to let the mobile user store the mobile video in the servers (let us remember that the trends in mobile phones are to allow the user to act easily with these kinds of servers: for example, currently it is possible a mobile user stores a mobile video in *YouTube* with a simple *click*) or even add an image in a location of the *Google Maps*. This type of procedure provides an easy-to-use system. In the experimental results shown in Section 5 we use our server to test this proposal and we are researching the way to upload the tagged videos to public servers.

## 4. Analytical Model

Our first step has been to model the time needed to perform all the processes carried out in our system in order to test if it will provide a good service for the end users. First, it is necessary to assess the time required for the system from the video capturing and tagging until its storage in Internet servers. This time should be analyzed in order to see how much time is required since we started recording a video until the video can be accessed by another user using the Internet. The total time *T* is given by Equation (1):

(1) $$T=t_{\mathrm{sensing}}+t_{\mathrm{processing}}+t_{\mathrm{TX}}+t_{\mathrm{propagation}}+t_{\mathrm{RX}}+t_{\mathrm{saving}}+t_{\mathrm{presentation}}$$

where *t_sensing_* is the time required to gather the data from the embedded sensors. It is the time of the most delayed sensor data. The highest time is the time needed to scan the values of the GPS (*T_GPS_*), as we will see in the next section. So, bearing this in mind, we can affirm that *t_sensing_* ≅ *T_GPS_*, which is around 1 second. *t_processing_* is the time required by the device mobile to read the tag from the sensor and to tag the video frames. In this case *t_processing_* = *T_RSen_* + *T_Tag_*. According to our measures shown on the next section, the value will be around 50 ms. *t_TX_* is the time needed to encapsulate all data into the IP packet in order to send the information through several networks. *t_RX_* is the time required to decapsulate the data. Generally *t_TX_* is equal to *t_RX_*. Nowadays these times are very low, because they depend on features of the end devices and these devices have high performance. *t_propagation_* is the time needed to send all the data via the interconnected networks to the Internet server. It depends on *t_RQ_* (the request time) and the propagation time of each network. Bearing in mind that the propagation time in a network (*Delay_i_*) may vary depending on the amount of traffic in the network, we have added the instantaneous efficiency of each network (*φ_i_*). Hence, the propagation time, when there are *n* networks between the mobile phone and the server, is shown in Equation (2):

(2) $$t_{\mathrm{propagation}}=t_{\mathrm{RQ}}+\sum_{i=1}^{n}\varphi_{i}\cdot {\mathrm{Delay}}_{i}$$

Finally, we have added *t_saving_* and *t_presentation_* to Equation (1). These times depend on the servers, which accept the tagged videos sent by the mobile devices. But, the significant time is *t_saving_*, because *t_presentation_* is negligible when the data have been stored on the servers. In order to analyze the parameter *t_saving_* we have used an approximation of GI/G/m model [47]. Firstly, we assume that the arrival process is given by the arrival rate *λ* and the squared coefficient of the variation of an interarrival time ( $c_{a}^{2}$). Moreover, the service-time distribution is specified by *τ* and its squared variation coefficient ( $c_{s}^{2}$). The next description of this model thus depend on the 4-tuple ( $\rho ,c_{a}^{2},c_{s}^{2},m$) where *ρ* = *λτ*/*m. t_saving_* can be estimated by *t_saving_* = *EW* + *τ*, where *EW* is the expected waiting time. Following the study performed in [47] we can extract an approximation for *EW* when $c_{a}^{2}=c_{s}^{2}=c^{2}$, which is shown in Equation (3).

(3) $$\mathrm{EW}(\rho ,c^{2},c^{2},m)\approx \Psi (c^{2},m,\rho )\cdot c^{2}\cdot \mathrm{EW}(M/M/m)$$

*EW* (*M*/*M*/*m*) is defined in Equation (4) and *Ψ* (*c*^2^, *m*, *ρ*) in the Equation (5).

(4) $$\mathrm{EW}(M/M/m)=\frac{\tau \left(\rho^{\sqrt{2(m+1)}+1}\right)}{m(1-\rho )}$$

(5) $$\Psi (c^{2},m,\rho )=\{\begin{array}{cc} 1 & c^{2}\geq 1\\ \phi_{4}{\left(m,\rho \right)}^{2(1-c^{2})} & 0\leq c^{2}\leq 1 \end{array}$$

where *ϕ*_4_ (*m*, *ρ*) can be estimated using the set of expressions shown in Equation (6):

(6) $$\begin{array}{c} \phi_{4}(m,\rho )=\text{min}\left\{1{,}^{\left(\phi_{1}(m,\;\rho )+\phi_{3}(m,\rho )\right)}/2\right\}\\ \phi_{1}(m,\rho )=1+\gamma (m,\rho ),\;\text{with}\;\gamma (m,\rho )=\text{min}\left\{0.24,(1-\rho )(m-1)\frac{\sqrt[{2}]{4+5m}-2}{16\mathrm{mp}}\right\}\\ \phi_{2}(m,\rho )=1-4\gamma (m,\rho )\\ \phi_{3}(m,\rho )=\phi_{2}(m,\rho )\cdot e^{\frac{-2(1-\rho )}{3\rho }} \end{array}$$

When $c_{a}^{2}\neq c_{s}^{2}$, *EW* can be expressed by Equation (7):

(7) $$\mathrm{EW}(\rho ,c_{a}^{2},c_{s}^{2},m)\approx \phi (\rho ,c_{a}^{2},c_{s}^{2},m)\cdot \left(\frac{c_{a}^{2}+c_{s}^{2}}{2}\right)\cdot \mathrm{EW}(M/M/m)$$

where *EW* (*M*/*M*/*m*) is defined in Equation (4) and *ϕ*( $\rho ,c_{a}^{2},c_{s}^{2},m$) in Equation (8):

(8) $$\phi (\rho ,c_{a}^{2},c_{s}^{2},m)=\{\begin{array}{ll} \left(\frac{4(c_{a}^{2}-c_{s}^{2})}{4c_{a}^{2}-3c_{s}^{2}}\right)\phi_{1}(m,\rho )+\left(\frac{c_{s}^{2}}{4c_{a}^{2}-3c_{s}^{2}}\right)\Psi ((c_{a}^{2}+c_{s}^{2})/2,m,\rho ) & c_{a}^{2}\geq c_{s}^{2}\\ \left(\frac{c_{s}^{2}-c_{a}^{2}}{2c_{a}^{2}+2c_{s}^{2}}\right)\phi_{3}(m,\rho )+\left(\frac{c_{s}^{2}-3c_{a}^{2}}{2c_{a}^{2}-2c_{s}^{2}}\right)\Psi ((c_{a}^{2}+c_{s}^{2})/2,m,\rho ) & c_{a}^{2}\geq c_{s}^{2} \end{array}$$

Using the data presented on [47] we have created Table 1 where we compare between exact waiting time and the expected waiting time calculated with previous equations. In this case we have selected a G/H2/m distribution with $c_{s}^{2}=9.0$. In this table we present several results for different traffic intensities and arrival process variability parameters. We can observe that the more number of servers the lower waiting time. Moreover, the higher traffic intensity, the higher waiting time.

**Figure 7.**
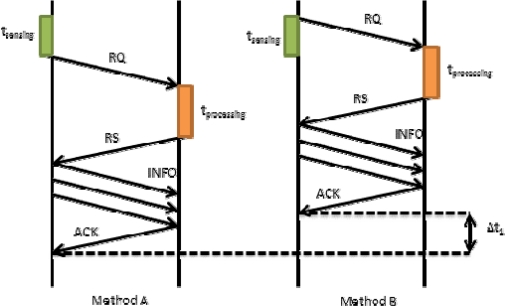
Request process comparison with short t_sensing_.

In Equation (2) we have seen that *t_propagation_* also involves *t_RQ_*. This time may affect to the final time in our proposal. Figure 7 shows how *t_RQ_* affects the request process when there is a short sensing time. On the left we show Method A, in which the device firstly senses the data and then sends a request packet (RQ) to the server. Finally, the server processes the request and sends a reply. If this reply is OK, the mobile device will send the tagged video to the server. In this case, the request time is *t_RQ_Meth_A_*. On the right we show our proposed method (Method B). It allows improving the system in terms of time. The mobile device sends the RQ packet immediately after starting the sensing process. Then, the system follows the regular designed steps. We can observe that request time is shorter. In this case, the request time is given by Equation (9):

(9) $$t_{\mathrm{RQ}\_\mathrm{Meth}\_B}=t_{\mathrm{RQ}\_\mathrm{Meth}\_A}-\Delta t_{1}$$

**Figure 8.**
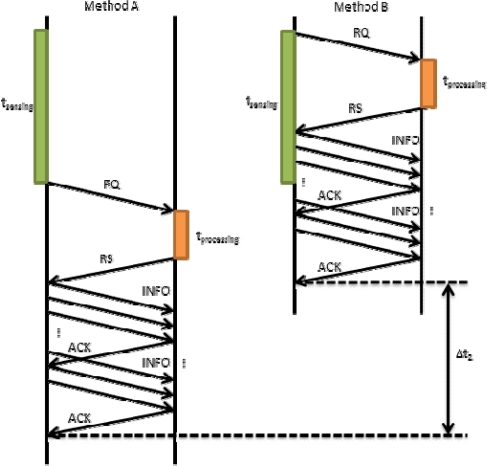
Request process comparison with higher t_sensing_.

In Figure 8, we performed the same comparison, but in this case with higher *t_sending_*. In this figure we see that the improvement is higher than in Figure 7 (Δ*t*_2_ ≫ Δ*t*_1_). According to this data we can affirm that if we use method B, regardless of *t_sending_*, we will obtain a shorter *t_RQ_*. Moreover, this improvement, Δ*t*, is directly proportional to *t_sending_*.

**Figure 9.**
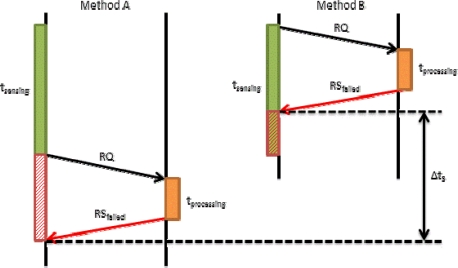
Failed request process comparison with high t_sensing_.

When the request process is denied, because the server cannot store the data for any reason, Method B continues improving the system. Figure 9 shows what happens in both cases when a mobile device sends a RQ message and the server replies with an RS_failed_ message. In method A, we see that the RQ message is sent after sensing, so the device stores the data until it receives the RS_failed_ to perform the appropriate task (store the tagged video on a file or delete the tagged video). In method B, the mobile device sends the RQ message when it starts the sensing process, so the system receives the RS_failed_ before completing the sensing process. In this case, we have considered two options. The first one lets the user to stop the process and delete the data, and the second process lets the user continue sensing and storing the tagged video locally.

**Figure 10.**
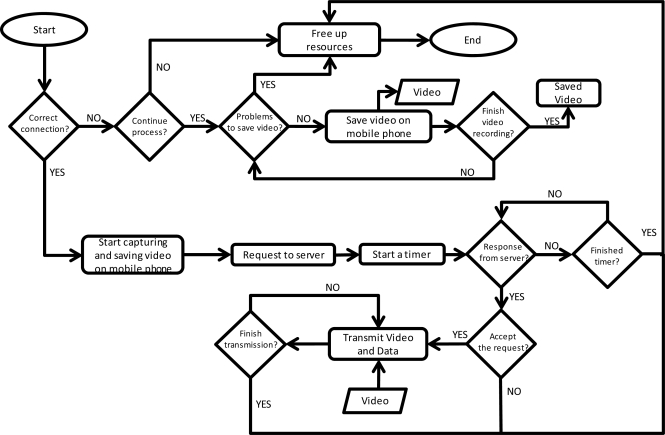
Algorithm for improving the request process.

Figure 10 shows the algorithm used to improve the request process. The explanation of the designed algorithm is as follows. The first step performed by the device is to check if it has an appropriate connection to perform the task. If the connection has not enough available bandwidth, the system lets the user to decide to continue or end the process. If the user chooses to continue, the device will store the tagged video in memory and the storage process will be controlled continuously (in order to check the available memory). The stored video can be sent to the server when there device has better connection. When the device checks that it has enough available bandwidth it begins the video capture and tagging processes. Then, a RQ packet is sent to the server and a timer is activated. This timer is used to check the reply of the server. If the mobile device does not receive a reply, it will finish the capturing process or stores the tagged video in memory. If the server replies, the device will start the transmission of the tagged video till the capture is finished.

## 5. Performance Evaluation of Tagging and Transactions

We have performed several experiments in order to test the performance of our mobile application. In particular, we have evaluated the tagging process and the communication performance between the mobile phone and the Server. We have used a HTC Tattoo mobile phone to experimentally show the viability of our proof of concept. The technical characteristics of the mobile phone are presented in Table 2.

### 5.1. Experimental Performance Evaluation of the Tagging Software

In this section we present the experimental results after measuring the next parameters: *T_GPS_*, *T_RGPS_*, *T_Sen_*, *T_Rsen_*, *T_Tag_* and *T_Tot_*. For the values presented in this section we repeated the experiments several times obtaining for all the measurements similar values (low typical deviation).

**Figure 11.**
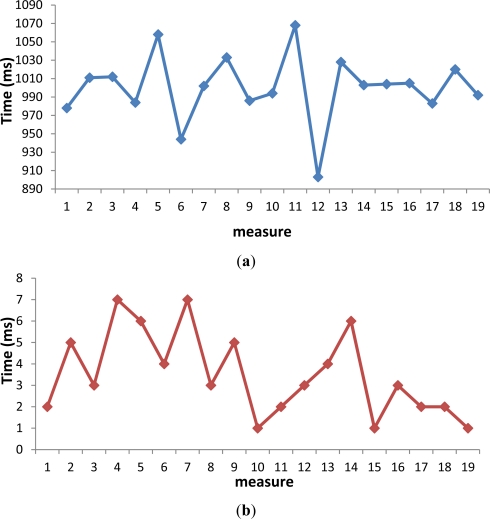
(**a**) Time needed to scan the values of the GPS (*T_GPS_*); (**b**) Time needed to read the value of the GPS (*T_RGPS_*).

We measured the values of *T_GPS_* using a satellite (we do not use the combined method of GPS and WiFi). The minimum time spent in scanning the GPS value was 903 *ms* and the maximum value was 1,068 ms. The mobile phone scanned different sets of 19 values with similar results. The average value was 1,000 ms. That is, the time needed to scan the GPS value is 1 s. This is a bigger value than 40 ms. This means some images must be tagged with the same GPS value. The *T_RGPS_* is insignificant compared with the *T_GPS_* due to its values are in the interval from 1 ms to 7 ms, with an average value of 3.5 ms. In Figure 11 (a,b) are shown these sets of values.

**Figure 12.**
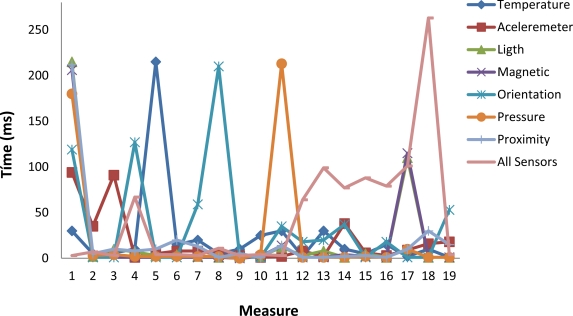
Time needed to read the values of the sensors (*T_sen_*).

In Figure 12 we show the difference between the values of *T_Sen_* measured in two consecutive reads for the following sensors: temperature, accelerometer, orientation, light, magnetic, pressure and proximity. In addition we also show the time needed to read simultaneously all the sensors. Some interesting points must be highlighted: The major values are concentrated in the interval from 1 ms to 30 ms. That means the latency of the measurement of consecutive values is in the normal case very low. We also show that there are several values in the approximate interval from 100 ms to 120 ms. This means that in several cases the latency is around 115 ms what means that consecutive images must be tagged with the same value of the sensor. Only a few values are greater than 150 ms and lower than 220 ms. When all the sensors are read the same shape of the curve is obtained. But in this case a very high value is found for the 18^th^ measure. The conclusion is that the Android software introduces a variable time to read the sensors which implies to repeat the value of the sensor for several consecutive images.

**Figure 13.**
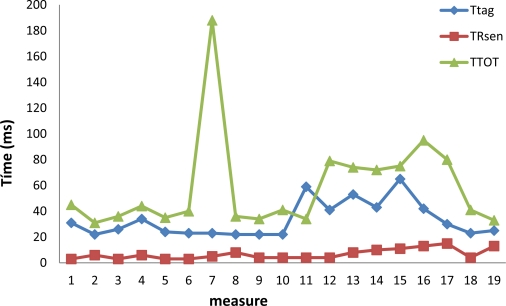
Time needed to tag the video frames.

The values *T_Rsen_* are very small in all cases as is shown in Figure 13. The values of *T_Tag_* are normally ten times higher than the values of *T_Rsen_* (with singular cases). The values of *T_Tot_* are always in the interval from 40 ms to 80 ms but a singular value is found for the 8th measure. These values mean that in general the values needed to tag the video frames and store them in the SD card are acceptable.

The experimental results of tagging in the mobile phone indicated that it is possible to tag mobile videos in the mobile phone in real time. Moreover, the overhead introduced by our tagging software is relatively low.

### 5.2. Performance Evaluation of the Transactions with the Internet Video Server

As mentioned before, the application allows users to share videos tagged with others through a server platform. The video upload from the mobile phone has been tested using a WiFi connection because it is more and more usual to find WiFi hotspots offering WiFi connectivity at no additional cost. Our proposal does not depend on the wireless technology used to send the videos, and it can be used in 3G technology as well, because the upload rate could even be up to about 10 Mbps in some operators. A detailed performance test about the bandwidth needed for video delivery over several wireless technologies is shown in [6].

Taking into account that the user sends a multimedia container file with the tag and the video and taking into account that this multimedia container is approximately 1 MB (considering video files of 10 seconds stored in the mobile phone), one can stay that for a connection with a speed of 11 Mbps, there will be not problem to send the files to the server as regards bandwidth availability.

The delay *jitter* of packets (*PDV*, *Packet Delay Variation*) in communication networks is defined as the difference in point to point delays, among several packets selected from a data stream, where losses are ignored. This definition is taken from the *Request For Comments* (*RFC*) 3393 [48], which explains this phenomenon, sometimes incorrectly named as jitter [49]. In fact, the aforementioned RFC specifies that the term is not used because it is considered in a different manner and by different groups of people, which would cause confusion.

PDV is the packet delay time from the start of transmission at the source, from the first byte of the packet until the last byte is received at the destination. To calculate the PDV, the delay components that do not vary can be overlooked. For example, if the packet size is the same and the transmission time and receiving time in the data buffers do not vary, the contribution of the time (the one the packet takes to cross the network to reach the destination buffer) will only be taken into account.

**Figure 14.**
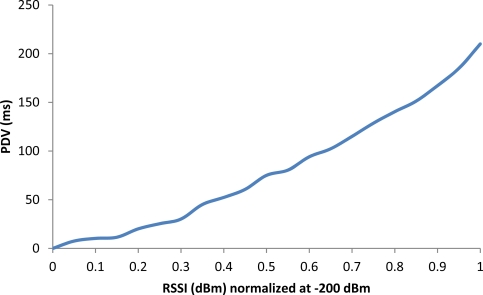
Packet delay variation *versus* RSSI.

The most important factors that affect the PDV are undoubtedly congestion or channel bit rate (due to the noise, multipath, mobile phone is far from the WiFi Access Point, intermittent disconnections...). This leads to packet losses or to a relatively large difference between the time of transmission of a packet and the time of receipt. If there are no disconnections, when the delay of packets increases, the PDV will increase. Thus, if a user is downloading a file, the waiting time will increase proportionally to the increase of PDV. On the contrary, if disconnections occur, unless there is an attention mechanism to them, we must forward the video and its tagging file again starting from scratch. In this case, the time the user must wait will increase, but the delay between packets will depend only on the channel conditions. The variation of packet delay grows exponentially as the RSSI decreases its value, which corresponds to a WiFi signal level received less, and therefore slows down the operation of the application (Figure 14).

We have measured the PDV both communicating only the video and communicating the tagged video in order to make a comparison between the results obtained in both cases. For a given level of RSSI (weak or strong), we have considered the following parameters to measure the communicating time (delay) extreme-to-extreme:

- *T_I_*: Time spent by the current packet to be transmitted
- *T_I-1_*: Time spent by the previous packet to be transmitted
- *N*: Number of packets that are considered in the measurement. They correspond to time interval.

**Figure 15.**
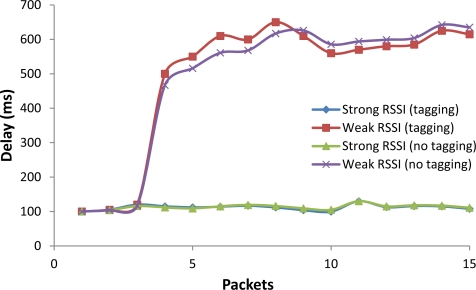
Delay of packets both for tagging and non-tagging videos.

**Figure 16.**
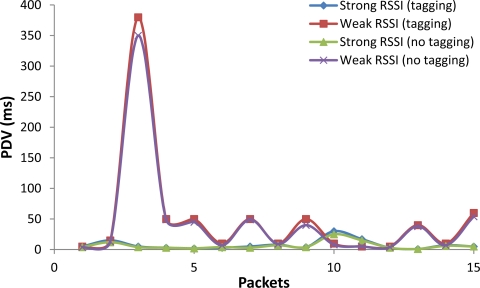
PDV in tagging and non-tagging videos.

The delay (Figure 15) and PDV (Figure 16) were measured taken into account current and previous packets in the given interval of time. We have measured the delay and PDV in the WiFi access network allocating the Internet Video Server in a computer directly connected to a WiFi/Ethernet router.

Figure 15 shows the delay of packets. Let us analyze the case of Weak RSSI. The three first packets had low delay but it increased for packet fourth to 500 ms. Then the delay maintained a constant interval of values around 500 ms and 650 ms. The differences between tagged and non-tagged video are minimal. We have obtained low delay values for the case of Strong RSSI (the average delay was around 100 ms). The case of Strong RSSI is the optimal one since the mobile phone is well connected to the Access Point, no packet loss, no intermittent disconnections…. Again, the differences between tagged and non-tagged video were minimal.

In Figure 16 are shown the values of PDV for Weak and Strong RSSI cases. For Weak RSSI, the PDV for the third packet is around 350 ms. The PDV for the next packets is small. This pattern is practically the same for tagged and non-tagged video packets. The average PDV for tagged video packets was 50 ms, and the average PDV for non-tagged packets was 45.5 ms. For Strong RSSI, the PDV was very low for both tagged and non-tagged videos (the average PDV is 7.5 and 6.2 ms respectively).

The values obtained for delay and PDV indicate that the real time communication of tagged videos is possible and with low PDV for a WiFi (version g) channel. Moreover, the tagging of videos we introduced produced a very low overhead in communications.

## 6. Conclusions

Current mobile smart mobile phones have very powerful video cameras. They also have several sensors that can be used to tag the mobile video. The processing power of current smart mobile phones is very high. This make possible to implement mobile platforms like Android over 1 GHz processors that have up to 256 MB RAM. They also have very high speed wireless communication cards like WiFi (version g).

In this paper we have presented an application that records and tags the video frames with data collected by the Android mobile phone’s sensors. Tagged videos are uploaded to an Internet video server and accessible for users. The experimental results show that is possible to tag mobile video frames in real time and using the WiFi wireless card interface of the mobile phone is also possible to make real time transactions with the video Server that hosts the videos.

The next version of mobile communication (*Long Term Evolution*) will exhibit transmission speeds of about 50 Mbps. This means that the tests we have implemented using WiFi could be used in these kinds of mobile communication technologies naturally. We modestly think that this is a starting work that has opened the door to new kind of mobile video applications in the close future. We think this means that our solution could be very successful in the near future.

As part of the future work we will propose to a Telco operator the possibility to implement our mobile application in their mobile transport network. We also will work in the definition a semantic searching application of mobile semantic videos in the Video Server. Integrating this application with our current mobile application it will be possible a mobile user to take a short video from a determined place, to tag it, to send it to the Internet Video Server (a public one such as *YouTube*). Then another mobile user could make a semantic video search in the Internet Video Servers. These searches can be done in Real Time. On the other hand the semantic video searches can also be done in tagged videos stored in the mobile phones. This fact confers to our system the typical characteristics of a semantic wireless sensor web considering the mobile phones as high powerful wireless sensors.

## Figures and Tables

**Table 1.** Comparison between exact waiting time and EW in G/H2/m with $c_{s}^{2}=9.0$.

**Number of server (*m*)**	**Traffic intensity (*ρ*)**	**Method**	**Arrival process variability parameter**		
			$c_{a}^{2}=0.5$	$c_{a}^{2}=2.0$	$c_{a}^{2}=9.0$
2	0.60	Exact	2.20 s.	2.86 s.	5.28 s.
		[47]	2.13 s.	2.65 s.	5.06 s.
	0.80	Exact	7.79 s.	9.46 s.	16.47 s.
		[47]	7.69 s.	9.16 s.	16 s.
	0.90	Exact	19.5 s.	23.1 s.	39 s.
		[47]	19.4 s.	22.7 s.	38.4 s.
4	0.60	Exact	0.56 s.	0.82 s.	1.82 s.
		[47]	0.63 s.	0.80 s.	1.61 s.
	0.80	Exact	3 s.	3.79 s.	7.15 s.
		[47]	3.12 s.	3.75 s.	6.71 s.
	0.90	Exact	8.69 s.	10.5 s.	18.3 s.
		[47]	8.82 s.	10.4 s.	17.7 s.

**Table 2.** Technical characteristics of the HTC Tattoo mobile phone.

**Processor**	Qualcomm® MSM7225™, 528 MHz
**Memory**	- 512 MB of Read Only Memory (ROM) - 256 MB of Random Access Memory (RAM) - microSD™ memory card (SD 2.0 compatible)
**Display**	240 × 320 Quarter Video Graphics Array (QVGA)
**Data Network Interface Cards**	- High Speed Downlink Packet Access/Wideband Code Division Multiple Access: 384 kbps up-link and 7.2 Mbps down-link - Enhanced Data Rate Bluetooth® 2.0 - Wi-Fi®: IEEE 802.11 b/g
**Video color camera**	- 3.2 Mpixels - Video supported formats: MPEG-4, H.263, H.264 and Windows Media® Video 9
**Battery life time**	- Up to 342 minutes for WCDMA (talk time) - Up to 520 hours for WCDMA (standby time)
**Android platform**	1.6
